# Combined Ultrasound and MRI Assessment in Patients Undergoing Reoperation for Recurrent Papillary Thyroid Carcinoma: Oncological Outcomes and Surgical Safety

**DOI:** 10.3390/curroncol33020098

**Published:** 2026-02-04

**Authors:** Zimei Tang, Jie Liu, Rong Wang, Gang Tian, Anwen Ren, Jiexiao Li, Yiran Wang, Wen Yang, Peng Sun, Tao Huang, Ximeng Zhang, Jie Ming

**Affiliations:** 1 Department of Breast and Thyroid Surgery, Union Hospital, Tongji Medical College, Huazhong University of Science and Technology, 1277 Jiefang Road, Wuhan 430022, China; d202382108@hust.edu.cn (Z.T.); m202175949@hust.edu.cn (R.W.); m202276043@hust.edu.cn (G.T.); 202562000973@email.sdu.edu.cn (A.R.); d202282036@hust.edu.cn (J.L.); m202476355@hust.edu.cn (Y.W.); yangwenwh@hust.edu.cn (W.Y.); huangtaowh@hust.edu.cn (T.H.); 2 Department of Radiology, Union Hospital, Tongji Medical College, Huazhong University of Science and Technology, 1277 Jiefang Road, Wuhan 430022, China; 2013xh0830@hust.edu.cn; 3 Hubei Provincial Clinical Research Center for Precision Radiology & Interventional Medicine, Wuhan 430022, China; 4 Hubei Province Key Laboratory of Molecular Imaging, Wuhan 430022, China; 5 Clinical & Technical Solutions, Philips Healthcare, Beijing 100600, China; peng.sun@philips.com

**Keywords:** reoperation, papillary thyroid carcinoma, magnetic resonance imaging, ultrasound, neck dissection, lymph node metastasis, recurrence-free survival

## Abstract

Reoperation for papillary thyroid carcinoma (PTC) requires precise lymph node metastasis assessment, yet ultrasound (US) alone may be insufficient in complex or high-risk cases. This study evaluated whether supplementing US with magnetic resonance imaging (MRI) improves surgical guidance and outcomes in reoperation. We retrospectively analyzed 375 patients who underwent reoperation between 2014 and 2022. Propensity score matching yielded 101 patients in the USUS-only group and 62 in the US+MRI group. Pathological and imaging data were compared to assess diagnostic performance, surgical outcomes, biochemical responses, and recurrence-free survival. The combined approach significantly increased sensitivity for detecting central lymph node metastasis from 52.5% to 90.9% and resulted in higher rates of central neck dissections (65.1% versus 45.5%) with greater lymph node yield (median: 29 versus 20) but lower lymph node ratios. More patients in the combined group achieved excellent biochemical responses (50.0% versus 27.7%). While overall recurrence-free survival (RFS) was not significantly different, the US+MRI group showed improved RFS among patients with ≥2 positive central nodes (HR = 0.24, *p* = 0.032). Importantly, complication rates were comparable, suggesting that improved outcomes were achieved without added surgical risk. Combined US and MRI assessment enhances diagnostic performance and may improve surgical and oncological outcomes in select high-risk patients undergoing PTC reoperation.

## Simple Summary

Papillary thyroid cancer is the most common type of thyroid cancer, and it is sometimes recurrent after initial treatment, requiring reoperation. Ultrasound is typically used to assess whether cancer has spread to nearby lymph nodes before operations, but it may miss some cancerous lymph nodes when not paired with another form of assessment. This study explored whether supplementing ultrasound with magnetic resonance imaging could improve surgical planning and outcomes in patients undergoing reoperation. The results showed that the combined assessment significantly helped in detecting more involved lymph nodes, leading to more accurate surgical targeting and improved treatment response, without increasing complication rates. These findings suggest that while MRI is not needed for all patients, it may provide added value in selected high-risk cases, helping surgeons perform safer and more effective reoperations.

## 1. Introduction

Papillary thyroid carcinoma (PTC) generally has an excellent prognosis [1]. However, recent cohort studies have reported recurrence rates ranging from approximately 8% to 28%, depending on risk stratification and follow-up duration. However, recent cohort studies have reported recurrence rates ranging from approximately 8% to 28%, depending on risk stratification and follow-up duration [2,3]. Even in low-risk cases such as papillary thyroid microcarcinoma (PTMC), the long-term recurrence rate may approach 3% [4]. Among these recurrent cases, locoregional lymph node metastasis (LNM) is the most common pattern, which may increase the risk of distant metastasis and affect disease-specific survival [5,6]. For patients with locally advanced PTC, timely reoperation is essential to prevent tumor invasion of neighboring structures (such as the trachea, esophagus, or mediastinal vessels). However, even with reoperation, complete remission is not always achieved [7,8]. Many patients needing reoperation show symptoms at first follow-up, making compartment-oriented neck dissection essential to prevent more recurrence or persistence [9]. Therefore, comprehensive preoperative imaging is crucial for accurately localizing the involved compartment to guide effective surgical intervention.

Ultrasound (US) is the primary imaging modality for diagnosing PTC, but solely relying on US may lead to incomplete assessment, especially in reoperative settings, where access to the central and mediastinal areas is limited due to scar tissue and anatomical alterations [10]. The 2025 American Thyroid Association differentiated thyroid cancer guidelines recommend adjunctive cross-sectional imaging, such as CT or MRI, when US findings are inadequate [11]. A meta-analysis reported MRI’s sensitivity and specificity for PTC LNM to be 80% and 85%, respectively [12]. In comparison, AI-assisted US has shown a pooled sensitivity of 80–83%, outperforming conventional US, especially in complex regions like the central compartment [13]. Previous studies, including our own, have highlighted MRI’s superiority in assessing central LNM and extrathyroidal extension [14,15,16]. However, whether this translates into improved surgical quality and oncologic outcomes remains unclear.

To address this gap, our study investigates the impact of preoperative MRI on surgical outcomes in patients with recurrent or persistent PTC. Propensity score matching (PSM) was employed to minimize potential biases and ensure a balanced comparison of outcomes between the US-only and US+MRI groups, thereby providing a robust evaluation of MRI’s benefits in reoperation.

## 2. Materials and Methods

### 2.1. Patients and PSM

**Figure 1:**
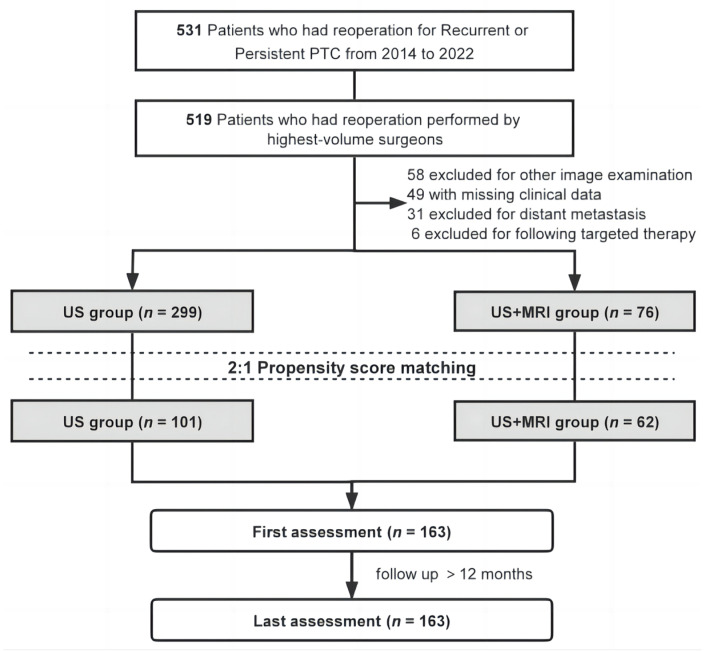
Flowchart of patients with recurrent or persistent papillary thyroid cancer (PTC) included in the study cohort.

Patients who underwent reoperation for recurrent or persistent PTC between 1 January 2014 and 31 December 2022 were screened (Figure 1). The inclusion criteria were: (1) total thyroidectomy performed; (2) preoperative neck US and/or MRI within three months before reoperation; (3) pathologically confirmed recurrent or persistent PTC; and (4) reoperation performed by a highly experienced surgeon (over 1000 thyroid procedures). The choice between undergoing US alone or US combined with MRI was based on patient preference or their doctor’s advice. The exclusion criteria were as follows: (1) missing data or lost to follow-up; (2) presence of distant metastasis or other primary malignancies; (3) patient underwent other neck preoperative imaging examinations; (4) patient received targeted treatment after reoperation. 

To minimize biases arising from variations in initial treatment characteristics and imaging modality selection, we employed propensity score matching for subsequent analyses. Variables denoting age, gender, body mass index (BMI), Hashimoto’s thyroiditis, primary operation type, tumor size, N stage, primary positive lymph nodes (PLNs), radioactive iodine (RAI), thyroglobulin levels, fine-needle aspiration (FNA) confirmation before reoperation, reoperation interval, and initial operation institutions were included in the PSM model (caliper = 0.02, ratio = 2:1) (Figure S1). FNA confirmation before reoperation was defined as the presence of ≥1 lymph node with cytologically confirmed malignancy.

### 2.2. Reoperation Strategy

Based on preoperative assessment, neck dissection (ND) included central neck dissection (CND) and lateral neck dissection (LND). Surgical specimens were removed and examined pathologically on a level-by-level basis. We analyzed the number of reoperative PLNs, reoperative lymph node yield (LNY), and reoperative lymph node ratio (LNR).

### 2.3. US and MRI Image Re-Evaluation

Two independent radiologists, blinded to patients’ preoperative pathology reports and outcomes, re-evaluated all MRI and US images from the US+MRI group in the PSM model. They recorded the location of suspicious lymph nodes (LNs) and their radiographic characteristics. Abnormal US findings suggestive of LNM included the following: round shape, irregular shape, loss of hilum, microcalcifications, cystic change, and peripheral vascularity [17]. LNs on MRI were considered malignant if they exhibited a round shape, necrosis or cystic change, extranodal extension, fusion, and exaggerated enhancement [18].

We focused on classifying metastatic LNs by level rather than by individual node or patient. Based on the AJCC 8th edition nodal classification scheme [19], the neck was divided into two compartments with seven levels, with the central compartment further divided into right and left sides. Each level was designated as benign or malignant based on the most suspicious LN. LNs involving two adjacent levels were assigned to the level encompassing the larger volume. Pathological and imaging data were analyzed level-by-level to determine the diagnostic value of different modalities (Figure S2).

### 2.4. Measures of Outcome

#### 2.4.1. Primary Outcome Measures

- Recurrence-Free Survival (RFS): RFS was monitored during follow-up, after reoperation. Recurrence or persistent disease was defined as locoregional lesions identified by fine-needle aspiration, reoperation, or distant metastasis confirmed by radiography.
- Biochemical Response: Biochemical response was assessed via serum thyroglobulin (Tg) and thyroglobulin antibody (TgAb) levels under TSH suppression treatment (Elecsys Tg II and Elecsys Anti-Tg kit, Roche Diagnostics GmbH, Mannheim, Germany).
- Response to Therapy Classification: Response to therapy classification was categorized according to the 2025 ATA guidelines, with potential categories being excellent response (ER), indeterminate response (IDR), biochemical incomplete response (BIR), and structural incomplete response (SIR).
- Postoperative complications: Postoperative complications were recorded according to clinical records and follow-up assessments. Hypoparathyroidism was defined as biochemical or symptomatic hypocalcemia requiring calcium or calcitriol supplementation, with permanent hypoparathyroidism defined as persistence beyond 6 months. Recurrent laryngeal nerve injury was classified as transient if voice dysfunction resolved within 6 months and permanent otherwise, based on laryngoscopic or clinical follow-up. Other complications (chyle leak, wound infection, hematoma, tracheal/esophageal injury) were defined by standard clinical criteria.

#### 2.4.2. Secondary Outcome Measures

- Extent of Reoperation: The involved compartment was determined by evaluation.
- Reoperative PLN, Reoperative LNY, and Reoperative LNR: Used as surrogates for the completeness of nodal compartment excision.
- Diagnostic Performance: Detection rates, sensitivity, specificity, positive predictive value (PPV), negative predictive value (NPV), and accuracy of US, MRI, and US+MRI were compared based on the diagnosis of nodal compartments.

### 2.5. Statistical Analysis

Statistical analyses were performed using SPSS v26.0 and R v4.2.1 software. Due to the skewed distribution of continuous variables, these data are reported as medians with interquartile ranges (IQRs), while categorical variables are presented as percentages and absolute numbers. The US+MRI group and the US group were compared using *t*-tests, non-parametric tests, or chi-square tests as appropriate. Standardized mean differences (SMD) of 0.2, 0.5, and 0.8 indicate small, medium, and large effect sizes, respectively. Survival outcomes were evaluated using the Kaplan–Meier method and log-rank test. A two-sided *p*-value of less than 0.05 was considered statistically significant.

## 3. Results

### 3.1. Patient Characteristics Before and After PSM

Table 1 summarizes the baseline clinical characteristics before and after PSM. In the overall cohort, 375 patients who underwent reoperation with US (*n* = 299) or US+MRI (*n* = 76) between 1 January 2014 and 31 December 2022 were identified. Before PSM, the US+MRI group had a higher proportion of male patients (36.84% vs. 25.75%) and a greater number of patients who did not undergo primary surgery at our institution (72.37% vs. 59.87%), though these differences were not statistically significant. After PSM, 163 patients were matched between the US group (*n* = 101) and the US+MRI group (*n* = 62).

For primary surgery, there were no significant differences in tumor size, N stage, or the number of positive lymph nodes between the US and US+MRI groups. After matching, there were no significant differences in baseline demographics or primary tumor-related characteristics between the two groups (SMD < 0.2).

### 3.2. Enhanced Lymph Node Detection with US+MRI

In the US+MRI group, LND was performed in 59 patients (42 unilateral; 17 bilateral), while CND was performed in 41 patients (24 unilateral; 17 bilateral). Metastatic LNs were identified in 150 (50.3%) of the 298 lateral levels: 37 (48.7%) at level II, 43 (56.6%) at level III, 52 (68.4%) at level IV, and 18 (25.7%) at level V. Of the 75 central levels, 37 (49.3%) were pathologically proven to have metastatic LNs. Overall, metastatic LNs were confirmed in 187 (50.1%) of the 373 dissected levels (Table S1).

**Figure 2:**
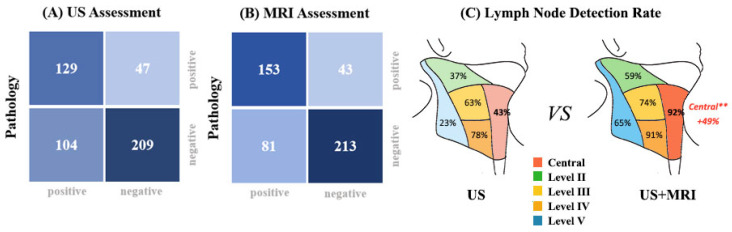
Confusion matrices showing diagnostic performance of (**A**) US alone and (**B**) combined US+MRI assessment. Each cell displays the number of total nodal compartments. Color intensity represents the absolute count. (**C**) Detection rate comparison between US alone and US+MRI across different neck nodal levels using pathological diagnosis as the gold standard. ** Combined US+MRI assessment improved the detection rate of central compartment lymph nodes by 49% compared to US alone.

Using pathological diagnosis as the gold standard, we evaluated the performance of each imaging modality (US, MRI, and US+MRI) by categorizing compartments with suspicious radiographic features as true or false positives (Table S2). Diagnostic performance for total nodal compartment disease is illustrated in Figure 2A,B and detailed in Table S3. US alone had a sensitivity, specificity, PPV, and NPV of 52.9%, 83.9%, 63.9%, and 68.4%, respectively, and MRI alone showed improved performance, with a sensitivity, specificity, PPV, and NPV of 64.2%, 85.5%, 81.6%, and 70.4%, respectively, particularly in detecting central LN metastasis. Notably, the combined US+MRI approach markedly increased sensitivity from 43.2% to 91.9% in the central compartment and from 55.3% to 71.3% in the lateral compartment (Figure 2C). Furthermore, it identified an additional 49 metastatic LN levels, significantly reducing the missed diagnosis rate for central LN metastasis by 48.6% (18/37) compared to US alone. Detailed analysis of radiologic–pathologic discordant cases is presented in Supplementary Table S4.

### 3.3. Enhanced Lymph Node Dissection Efficacy

Within the propensity score-matched cohort (Table 2), no significant differences were observed in focus size, thyroid bed recurrence, or extranodal extension between the groups. However, a greater proportion of patients in the US+MRI group underwent central neck dissection (65.1% vs. 45.5%, *p* = 0.018), while the distribution of lateral neck dissection remained similar. Notably, the median lymph node yield was significantly higher in the US+MRI group (29 vs. 20, *p* < 0.001), particularly in lateral neck dissection (24 vs. 18, *p* = 0.001). Although the number of metastatic lymph nodes removed was comparable (5 vs. 4, *p* = 0.218), the US+MRI group achieved a significantly lower LNR (0.14 vs. 0.24, *p* < 0.001), especially in central neck dissection (0.22 vs. 0.48, *p* = 0.019). These results indicate that integrating MRI with US leads to more comprehensive lymph node dissection during reoperation.

### 3.4. The Impact of US+MRI on Reoperation Outcomes for PTC

All patients in both groups were followed for a median of 36 months (IQR, 24–68 months) for the US group and 38 months (IQR, 24–64 months) for the US+MRI group. To evaluate the clinical benefits, comparisons were made at the first and last follow-up points. Both groups received similar radioactive iodine therapy after reoperation (Table S4).

#### 3.4.1. Biochemical Response to Reoperation

**Figure 3:**
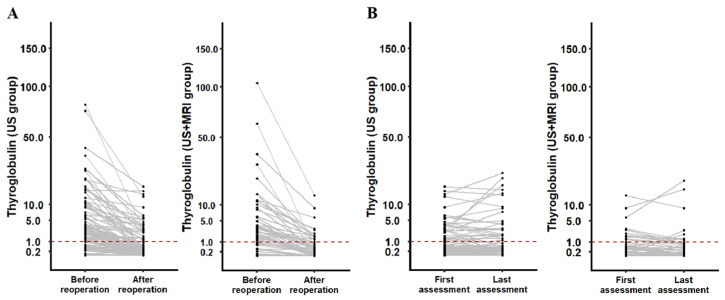
Changes in Tg (ng/mL) before and after reoperation (**A**) and changes in Tg in the first and last assessments after reoperation (**B**) in patients from the US group and US+MRI group in the propensity score matching model. Patients with Tg levels lower than 1 ng/mL (dotted lines) after reoperation are regarded as achieving biochemical complete remission.

Serum Tg levels significantly decreased in both groups after reoperation (*p* < 0.001), while the median Tg levels before reoperation were similar (2.21 ng/mL vs. 2.04 ng/mL, *p* = 0.466). Biochemical complete remission (BCR) was defined as Tg < 1.0 ng/mL. At the first assessment, 55.0% of patients in the US group and 65.2% in the US+MRI group achieved BCR (Figure 3A). At the last assessment, 55.0% in the US group and 69.6% in the US+MRI group achieved BCR (Figure 3B). Although the US+MRI group had lower Tg levels at both the first (0.14 ng/mL vs. 0.25 ng/mL) and last (1.11 ng/mL vs. 1.91 ng/mL) assessments, these differences were not significant. Overall, patients in the US+MRI group showed a more pronounced and sustained biochemical response.

#### 3.4.2. Response to Therapy Classification

Significant differences were observed in the distribution of response to reoperation between the two groups in the initial follow-up (*p* = 0.017, Table 3). In the US+MRI group, 50.0% achieved an excellent response, compared to 27.7% in the US group (*p* = 0.022). Several patients with severe locoregional disease maintained structural incomplete response (SIR) after reoperation (5% vs. 8%). During follow-up, some patients transitioned from an uncertain response (IDR and BIR) to a clear response (ER and SIR) in both groups. By the last assessment, the response distribution showed no significant difference between groups (*p* = 0.078), with the gap in ER proportions decreasing (58.1% vs. 37.6%, *p* = 0.11).

#### 3.4.3. Kaplan–Meier Curve Analysis

**Figure 4:**
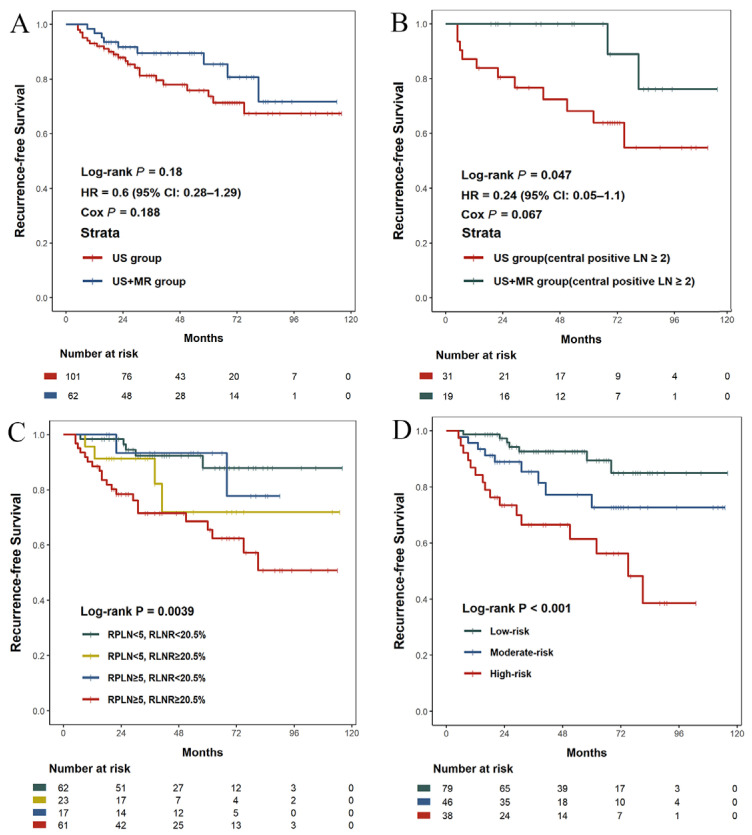
Kaplan–Meier curves of recurrence-free survival (RFS) in recurrent or persistent papillary thyroid cancer after reoperation. (**A**) US group vs. US+MRI group (*p* = 0.18). (**B**) US group vs. US+MRI group in patients with the number of central positive lymph nodes ≥ 2 (*p* = 0.047). (**C**) Four lymph node subgroups based on reoperative LNR and reoperative PLN (*p* = 0.0039). (**D**) Three risk subgroups (*p* < 0.001).

RFS rates were comparable between the overall US+MRI and US groups (14.5% vs. 22.8%), with no statistically significant difference observed (Log-rank *p* = 0.18; Figure 4A). However, subgroup analysis using Cox proportional hazards regression revealed that the survival benefit of MRI was significantly associated with the number of central positive lymph nodes (CPLN). Among patients with CPLN ≥ 2, those in the US+MRI group showed markedly improved RFS compared to those in the US group (Log-rank *p* = 0.047; Figure 4B), with a corresponding hazard ratio of 0.24 and a *p*-value of 0.067 in multivariate Cox regression analysis.

Further Cox regression analysis identified the reoperative LNR (*p* < 0.001), the number of reoperative PLN (*p* = 0.0028), and reoperative LNY (*p* = 0.096) as key prognostic indicators of RFS. Based on cutoff values of 20.5% for reoperative LNR and 5.5 for reoperative PLN, patients were initially categorized into four groups. Significant differences in RFS were observed among these groups (Log-rank *p* = 0.0039; Figure 4C). As Groups 1 (PLN < 5.5, LNR < 20.5%) and 3 (PLN ≥ 5.5, LNR < 20.5%) had similar survival outcomes, they were merged, resulting in a three-tier risk classification: low-risk (LNR < 20.5%, *n* = 79), moderate-risk (LNR ≥ 20.5% and PLN < 9, *n* = 46), and high-risk (LNR ≥ 20.5% and PLN ≥ 9, *n* = 38). RFS curves differed significantly among the three risk groups (Log-rank *p* < 0.001; Figure 4D).

### 3.5. Surgical Complications and Safety Outcomes

Despite more extensive dissection in the US+MRI group, complication rates were comparable after propensity score matching (Table 4). Permanent recurrent laryngeal nerve injury (2.9% vs. 3.2%, *p* = 1.000) and permanent hypoparathyroidism (3.9% vs. 4.8%, *p* = 0.707) showed no significant differences between groups. The overall complication rate (25.2% vs. 22.2%, *p* = 0.659) was consistent with reported rates for reoperative thyroid surgery and predominantly comprised transient complications that resolved with conservative management. No perioperative deaths occurred. One patient in the US+MRI group died 26 months after the operation from thrombotic complications during lenvatinib therapy, unrelated to surgery.

## 4. Discussion

Recurrent or persistent disease following initial treatment for PTC is common, frequently involving the lateral neck, central neck, and thyroid bed [20,21]. Although US is the standard imaging modality for assessing PTC LNM, its sensitivity for central LNM is limited (10–64%) due to interference from normal structures, scar tissue, and operator dependency [13]. In contrast, MRI offers superior soft-tissue contrast and, when combined with US, enhances the preoperative detection of metastatic LNs [22].

In our study, after propensity score matching (PSM), the US+MRI group showed markedly increased central LNM detection [23,24] and a higher central neck dissection rate (65.1% vs. 45.5%), without significant change in lateral dissection performance. In the lateral compartment, US and MRI showed similar diagnostic performance, which supports previous reports [25]. These findings show the value of MRI in enhancing compartment-oriented surgical planning. Our discordance analysis revealed that US typically failed to detect small, deep nodes, whereas MRI missed microscopic lesions or misclassified inflammatory changes. These patterns illustrate how combining both modalities helps overcome individual limitations in reoperative mapping.

This improved nodal detection translated into a higher median LNY (29 vs. 20, *p* < 0.001) and a lower LNR (0.31 vs. 0.42, *p* = 0.004), metrics that reflect more comprehensive nodal clearance. These quantitative improvements support the hypothesis that US+MRI facilitates more thorough and anatomically informed neck dissections.

We acknowledge that patients selected for US+MRI were more likely to have high-risk disease or ambiguous US findings, which may have influenced surgical decisions. While PSM reduced baseline imbalances, residual selection bias remains a limitation. Notably, the increased detection of metastatic nodes likely led to a more appropriate expansion of surgical extent, rather than indiscriminate aggressiveness. This highlights the role of enhanced imaging in tailoring reoperative strategy to individual disease burden.

Serum Tg levels remain a key biochemical marker for assessing treatment efficacy. Due to varying Tg cutoff values, comparing BCR rates across studies, which range from 19% to 71%, is challenging [26,27,28]. Although both groups experienced significant declines in Tg and similar BCR rates, the US+MRI group showed a higher excellent response rate at the initial assessment, with this therapeutic advantage persisting during follow-up. Interestingly, the differences in Tg level and response to therapy classification between the two groups narrowed over time, possibly due to adjuvant RAI effects in the US group and individual RAI responsiveness [29]. Nevertheless, the early Tg dynamics favoring the US+MRI group suggest more complete initial tumor clearance.

Notably, while overall RFS rates did not significantly differ between groups, subgroup Cox analysis revealed that patients with CPLN ≥ 2 significantly benefited from the addition of MRI, with a 76% risk reduction (HR = 0.24). This finding represents the first piece of evidence linking links MRI’s diagnostic improvement to meaningful long-term outcomes in a group of high-risk PTC patients.

Meanwhile, recent studies on surgical quality metrics in head and neck malignancies have demonstrated that a high LNR or low LNY signals suboptimal dissection [30,31]. Similar findings apply to PTC, where retrospective studies and SEER database analyses show that metrics like LNR, PLN, and LNY are associated with RFS or overall survival [32,33]. Consistent with these findings, our Cox regression analysis revealed that LNR and PLN are key factors for PTC recurrence after reoperation. Similarly, Yu et al. reported that a primary PLN of at least 32.74% and a primary LNY of at least 25 in LND are associated with poor RFS [34]. In our study, a PLN and LNR of at least 5.5 and 20.5%, respectively, in reoperation were linked to poor RFS.

Although some argue for less aggressive surgery due to PTC’s relatively indolent nature and low impact on mortality [35], this approach may be inappropriate for selected patients. Thorough and compartment-oriented ND remains critical in reoperation, not only to reduce recurrence and retreatment but also to alleviate the psychological burden of fear of recurrence [36], this approach may be inappropriate for selected patients. This underscores the critical importance of adequate surgery for achieving long-term outcomes, while the outdated practice of “cherry picking” is considered unreliable [37]. In real-world practice, reoperation decisions often relied on integrated imaging and biochemical assessment, and FNA-Tg was used selectively due to clinical urgency and technical/anatomical constraints. Nevertheless, the current guideline endorses FNA-Tg as the confirmatory gold standard when it may change surgical management [5]. Additional evidence is needed to better integrate FNA-Tg with imaging assessment in reoperation decision-making.

Our findings also suggest that MRI, when selectively used in patients with suspected central disease or bulky recurrence, may help achieve better oncological clearance while maintaining surgical safety. The postoperative hypocalcemia rate (transient: 6.67%, permanent: 2.67%) in our cohort was higher than that typically reported for primary thyroidectomy but consistent with published revision surgery series (permanent rate: 3–15%) [38]. For nearly all patients who had undergone at least unilateral CND during their initial surgery, these findings reflect the technical challenges of identifying and preserving parathyroid during reoperation, underscoring the importance of accurate assessment of CLNM and hypocalcemia risk before reoperation.

This study has several limitations. First, its retrospective design, limited number of patients, and follow-up duration contribute to unavoidable selection bias. Second, given 63.8% of initial surgeries were performed in outside institutions, variations in initial treatment and the lack of genetic mutation data [39] may have led to the omission of key prognostic factors. Third, while complication rates were recorded, laryngoscopic confirmation of nerve injury was not routinely performed, which may have led to underreporting [40]. Fourth, imaging interpretations were performed at a single institution and may not be generalizable across centers due to variable MRI quality or expertise. In addition, fine-needle aspiration thyroglobulin (FNA-Tg) was not routinely performed in all patients, partly due to anatomical inaccessibility or early institutional practices. Nevertheless, our data provide a compelling rationale to consider MRI as a selective adjunct to US in patients with suspicious central metastases or numerous metastatic nodes. Prospective multicenter trials are warranted to further validate these findings.

## 5. Conclusions

Our study demonstrates that the combination of MRI with US significantly enhances the detection of central LNM in patients undergoing reoperation for recurrent or persistent PTC. This improved detection was associated with more compartment-oriented surgery, better biochemical responses, and favorable trends in recurrence-free survival among high-risk patients. Larger, long-term studies are needed to confirm these findings and clarify the benefits of adding MRI to US in thyroid cancer surgery.

## Figures and Tables

**Table 1:** Baseline clinical characteristics of patients undergoing reoperation before and after propensity score matching.

Characteristics	Before Matching				After Matching			
	US Group (*n* = 299)	US+MRI Group (*n* = 76)	*p*-Value	SMD	US Group (*n* = 103)	US+MRI Group (*n* = 63)	*p*-Value	SMD *
Age (year, median [IQR])	37.67 [28.41, 46.38]	35.50 [28.06, 42.71]	0.418	0.113	35.58 [27.17, 44.92]	34.04 [27.40, 41.61]	0.718	0.057
Gender, *n* (%)								
Female	222 (74.25)	48 (63.16)	0.075	0.241	71 (70.30)	47 (75.81)	0.560	0.124
Male	77 (25.75)	28 (36.84)			30 (29.70)	15 (24.19)		
BMI (median [IQR])	23.00 [20.60, 25.45]	22.50 [21.17, 25.08]	0.999	0.011	22.30 [20.40, 25.20]	22.50 [21.12, 24.85]	0.697	0.067
Hashimoto’s thyroiditis, *n* (%)	87 (29.10)	20 (26.32)	0.736	0.062	30 (29.70)	17 (27.42)	0.893	0.051
Primary operation type, *n* (%)								
Total thyroidectomy	272 (90.97)	68 (89.47)	0.857	0.05	92 (91.09)	55 (88.71)	0.822	0.079
Lobectomy/Other	27 (9.03)	8 (10.53)			9 (8.91)	7 (11.29)		
Tumor size, *n* (%)								
≤10 mm	53 (17.73)	16 (21.05)	0.791	0.132	19 (18.81)	13 (20.97)	0.733	0.181
10–20 mm	103 (34.45)	28 (36.84)			35 (34.65)	22 (35.48)		
20–40 mm	112 (37.46)	24 (31.58)			41 (40.59)	21 (33.87)		
>40 mm	31 (10.37)	8 (10.53)			6 (5.94)	6 (9.68)		
Primary LN metastasis, *n* (%)								
N0	23 (7.69)	6 (7.89)	0.974	0.03	10 (9.90)	5 (8.06)	0.923	0.065
N1a	150 (50.17)	37 (48.68)			49 (48.51)	31 (50.00)		
N1b	126 (42.14)	33 (43.42)			42 (41.58)	26 (41.94)		
Primary PLN ≥ 5, *n* (%)	170 (56.86)	41 (53.95)	0.744	0.059	58 (57.43)	33 (53.23)	0.718	0.085
RAI before reoperation, *n* (%)	196 (65.55)	46 (60.53)	0.494	0.104	69 (68.32)	38 (61.29)	0.455	0.148
Tg before reoperation, *n* (%)								
<0.2	49 (16.39)	13 (17.11)	0.792	0.169	16 (15.84)	13 (20.97)	0.871	0.180
0.2–1	45 (15.05)	11 (14.47)			17 (16.83)	9 (14.52)		
1–5	102 (34.11)	31 (40.79)			38 (37.62)	25 (40.32)		
5–10	42 (14.05)	8 (10.53)			9 (8.91)	4 (6.45)		
≥10	61 (20.40)	13 (17.11)			21 (20.79)	11 (17.74)		
FNA confirmation before reoperation, *n* (%)	94 (31.44)	31 (40.79)	0.159	0.196	38 (37.62)	23 (37.10)	1.000	0.011
First reoperation, *n* (%)	241 (80.60)	59 (77.63)	0.676	0.073	83 (82.18)	49 (79.03)	0.771	0.080
Reoperation interval ≥1 year, *n* (%)	185 (61.87)	42 (55.26)	0.357	0.134	62 (61.39)	37 (59.68)	0.959	0.035
Initial operation institutions, *n* (%)								
WHUH	120 (40.13)	21 (27.63)	0.061	0.266	39 (38.61)	20 (32.26)	0.514	0.133
Others	179 (59.87)	55 (72.37)			62 (61.39)	42 (67.74)		

BMI: body mass index; LN: positive lymph node; PLN: positive lymph node; SMD: standardized mean difference. * SMD is the preferred way to describe imbalances in data that are descriptive of the sample. Standardized differences of 0.2, 0.5, and 0.8 correspond to small effect, medium effect, and large effect sizes, respectively. FNA: fine-needle aspiration; WHUH: Wuhan Union Hospital.

**Table 2:** Baseline clinical characteristics of patients undergoing reoperation in the propensity score matching model.

Variables After PSM	US Group	US+MRI Group	*p*-Value
Size of focus (median [IQR])	1.00 [0.67, 1.60]	1.10 [0.70, 1.60]	0.441
Thyroid bed recurrence, *n* (%)	9 (8.91)	4 (6.45)	0.791
Extranodal extension, *n* (%)	18 (17.82)	12 (19.35)	0.970
Central neck dissection, *n* (%)			
without	55 (54.46)	22 (34.92)	0.018
unilateral	21 (20.79)	24 (38.10)	
bilateral	25 (24.75)	17 (26.98)	
Lateral neck dissection, *n* (%)			
without	4 (3.96)	3 (4.84)	0.698
unilateral	63 (62.38)	42 (67.74)	
bilateral	34 (33.66)	17 (27.42)	
Reoperative LNY *, (median [IQR])			
central	5.50 [2.25, 10.75]	6.00 [4.00, 13.00]	0.279
lateral	18.00 [13.00, 27.00]	24.00 [17.00, 42.00]	0.001
Total	20.00 [13.00, 30.00]	29.00 [22.25, 45.75]	<0.001
Reoperative PLN *, (median [IQR])			
central	2.00 [1.00, 4.00]	1.00 [0.00, 3.00]	0.139
lateral	3.00 [1.00, 7.50]	3.00 [2.00, 5.50]	0.994
Total	4.00 [2.00, 9.00]	5.00 [3.00, 7.00]	0.218
Reoperative LNR *, (median [IQR])			
central	0.48 [0.25, 0.66]	0.22 [0.00, 0.50]	0.019
lateral	0.20 [0.08, 0.33]	0.13 [0.10, 0.24]	0.258
Total	0.24 [0.14, 0.38]	0.14 [0.10, 0.28]	0.004

* LNY (lymph node yield), PLN (positive lymph nodes), and LNR (lymph node ratio) refer to total counts across all compartments.

**Table 3:** Response to therapy classification in the propensity score matching model.

Disease Status	N (%) of Patients		*p*-Value *
	US Group	US+MRI Group	
After Reoperation: first assessment			
Excellent response	28 (27.72)	31 (50.00)	0.017
Indeterminate response	40 (39.60)	15 (24.19)	
Biochemical Incomplete response	28 (27.72)	11 (17.74)	
Structural Incomplete response	5 (4.95)	5 (8.06)	
After Reoperation: last assessment			
Excellent response	38 (37.62)	36 (58.06)	0.078
Indeterminate response	25 (24.75)	12 (19.35)	
Biochemical Incomplete response	15 (14.85)	5 (8.06)	
Structural Incomplete response	23 (22.77)	9 (14.52)	

* *p*-value determined using the chi-square test of independence.

**Table 4:** Postoperative complications of patients undergoing reoperation before and after propensity score matching.

Complications After Reoperation	Before Matching			After Matching		
	US Group (*n* = 299)	US+MRI Group (*n* = 76)	*p* Value	US Group (*n* = 103)	US+MRI Group (*n* = 63)	*p* Value
Hypoparathyroidism *, *n* (%)						
Transient (<6 months)	18 (6.02%)	7 (9.21)	0.296	11 (10.68)	5 (7.94)	0.554
Permanent (≥6 months)	7 (2.34)	3 (3.95)	0.429	4 (3.88)	3 (4.76)	0.780
Recurrent Laryngeal Nerve Injury, *n* (%)						
Transient (<6 months)	5 (1.67)	2 (2.63)	0.635	4 (3.88)	2 (3.17)	0.999
Permanent (≥6 months)	3 (1.00)	2 (2.63)	0.272	3 (2.91)	2 (3.17)	1.000
Bilateral injury	1 (0.33)	1 (1.32)	0.329	1 (0.97)	1 (1.59)	0.998
Wound infection, *n* (%)	12 (4.01)	4 (5.26)	0.621	5 (4.85)	3 (4.76)	1.000
Chyle leak, *n* (%)	3 (1.00)	1 (1.32)	1.000	2 (1.94)	1 (1.59)	1.000
Hematoma, *n* (%)	8 (2.68)	4 (5.26)	0.267	5 (4.85)	4 (6.35)	0.731
Tracheal/Esophageal Injury, *n* (%)	1 (0.33)	0 (0.00)	1.000	1 (0.97)	0 (0.00)	1.000
Total complication rate, *n* (%)	45 (15.05)	15 (19.74)	0.316	26 (25.24)	14 (22.22)	0.665

* hypoparathyroidism defined as calcium/vitamin D–dependent hypocalcemia.

## Data Availability

All raw datasets generated during and/or analyzed during the current study are not publicly available but are available from the corresponding author on reasonable request.

## Supplementary Materials

The following supporting information can be downloaded at https://www.mdpi.com/article/10.3390/curroncol33020098/s1, Figure S1: Proportion and distribution of propensity score in control group (US) and treated group (US+MRI) before and after matching; Figure S2: Pathological and image level-by-level analysis; Table S1: Number of nodal levels harboring metastasis among surgically dissected levels; Table S2: Nodal compartments detected at US, MRI, US+MRI of the resected specimen, along with the metrics of the diagnostic ability (pathological diagnosis was used as the gold standard method); Table S3: The diagnostic value of MRI features for central lymph nodal metastases; Table S4: Patterns of radiologic–pathologic discordance involving MRI in recurrent/persistent PTC reoperations; Table S5: number of radioactive iodine therapy after reoperation.

## Abbreviations

The following abbreviations are used in this manuscript:

BCR	Biochemical complete remission
BIR	Biochemical incomplete response
BMI	Body Mass Index
CND	Central neck dissection
ER	Excellent response
FNA	Fine-needle aspiration
IDR	Indeterminate response
IQR	Interquartile ranges
LN	Lymph node
LND	Lateral neck dissection
LNM	Lymph node metastasis
LNR	Lymph node ratio
LNY	Lymph node yield
ND	Neck dissection
NPV	Negative predictive value
PLN	positive lymph nodes
PPV	Positive predictive value
PSM	Propensity score matching
PTC	Papillary thyroid carcinoma
RAI	Radioactive iodine
SIR	Structural incomplete response
SMD	Standardized mean difference
Tg	Thyroglobulin
TgAb	Thyroglobulin antibody
US	Ultrasound
