# Supplementary material for: Combined Ultrasound and MRI Assessment in Patients Undergoing Reoperation for Recurrent Papillary Thyroid Carcinoma: Oncological Outcomes and Surgical Safety
Source: Curr Oncol. 2026 Feb 4;33(2):98. doi: 10.3390/curroncol33020098 (PMC12940060; doi:10.3390/curroncol33020098)
Supplement: Supplementary file 1 [file curroncol-33-00098-s001.zip › curroncol-4054441-supplementary.pdf]

Combined Ultrasound and MRI Assessment in Patients Undergoing Reoperation for Recurrent Papillary Thyroid Carcinoma: Oncological Outcomes and Surgical Safety

Zimei Tang [1,†], Jie Liu [2,3,4,†], Rong Wang [1], Gang Tian [1], Anwen Ren [1], Jiexiao Li [1], Yiran Wang [1], Wen Yang [1], Peng Sun [5], Tao Huang [1], Ximeng Zhang [1,*] and Jie Ming [1,*]

[1] Department of Breast and Thyroid Surgery, Union Hospital, Tongji Medical College, Huazhong University of Science and Technology, 1277 Jiefang Road, Wuhan 430022, China; d202382108@hust.edu.cn (Z.T.); m202175949@hust.edu.cn (R.W.); m202276043@hust.edu.cn (G.T.); 202562000973@email.sdu.edu.cn (A.R.); d202282036@hust.edu.cn (J.L.); m202476355@hust.edu.cn (Y.W.); yangwenwh@hust.edu.cn (W.Y.); huangtaowh@hust.edu.cn (T.H.)

[2] Department of Radiology, Union Hospital, Tongji Medical College, Huazhong University of Science and Technology, 1277 Jiefang Road, Wuhan 430022, China; 2013xh0830@hust.edu.cn

[3] Hubei Provincial Clinical Research Center for Precision Radiology & Interventional Medicine, Wuhan 430022, China

[4] Hubei Province Key Laboratory of Molecular Imaging, Wuhan 430022, China

[5] Clinical & Technical Solutions, Philips Healthcare, Beijing 100600, China; peng.sun@philips.com

* Correspondence: ximengzhangwh@hust.edu.cn (X.Z.); mingjiewh@hust.edu.cn (J.M.)

† These authors contributed equally to this work.

Supplementary Figures and Tables

Figure S1	pag. 2
Figure S2	pag. 3
Table S1	pag. 4
Table S2	pag. 4
Table S3	pag. 5
Table S4	pag. 6
Table S5	pag. 7

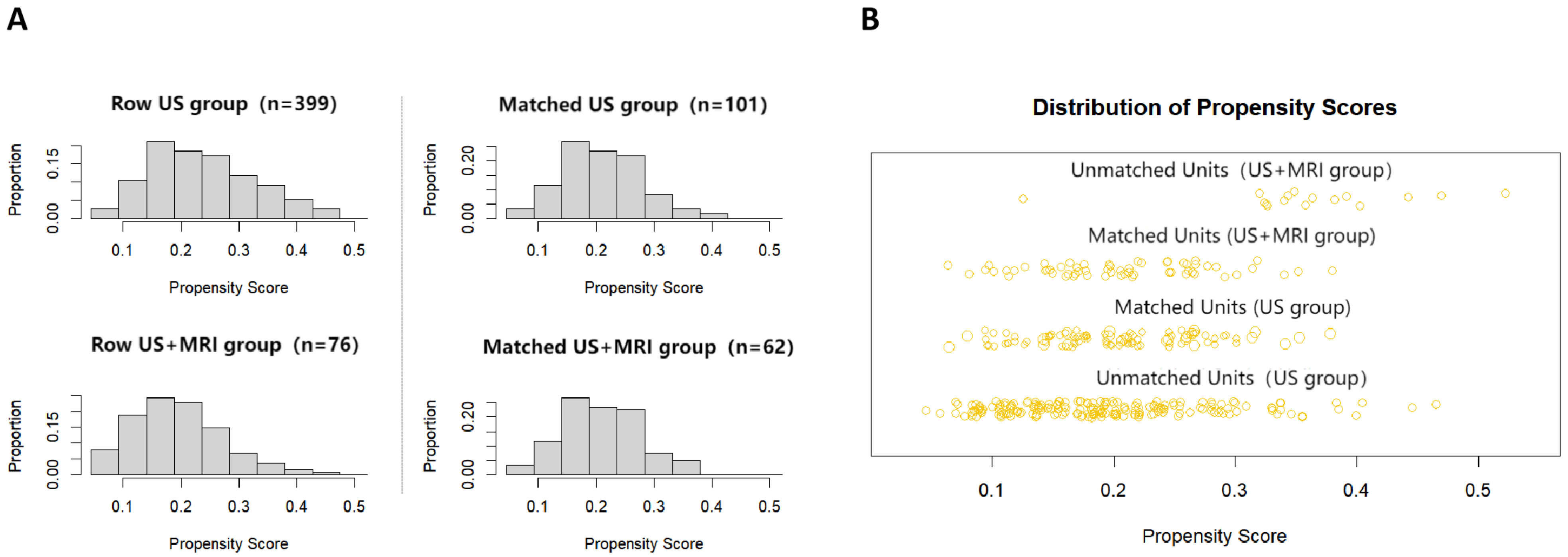

Figure S1. (**A**) Proportion and (**B**) distribution of propensity score in control group (US) and treated group (US+MRI) before and after matching.

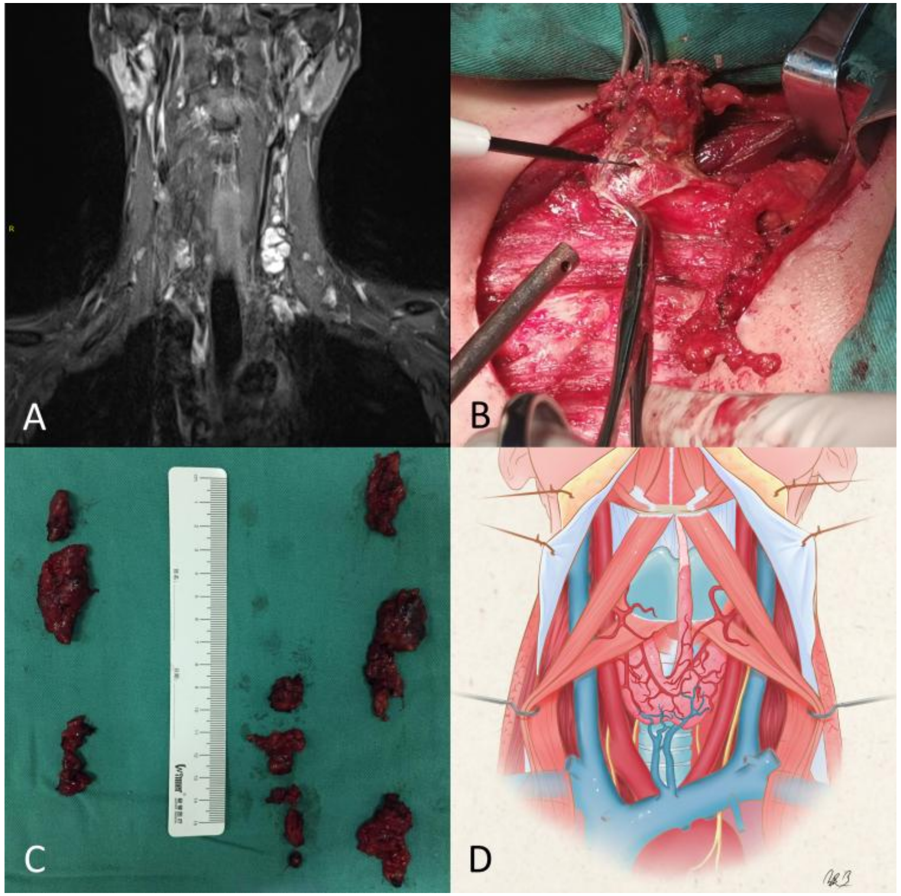

Figure S2. Pathological and image level-by-level analysis: (**A**) MR image of a lymph node with high suspicion for metastasis from PTC, (**B**) reoperation, (**C**) surgical specimens and (**D**) Schematic.

Table S1. Number of nodal levels harboring metastasis among surgically dissected levels.

Location	Surgically Dissected Nodal Levels	Positive (%)	Negative (%)
Lateral	396	189 (47.7%)	207 (52.3%)
II	102	49 (48.0%)	53 (52.0%)
III	103	54 (52.4%)	49 (47.6%)
IV	104	63 (60.6%)	41 (39.4%)
V	87	23(26.4%)	64 (73.6%)
Central	93	44 (47.3%)	49 (52.7%)
Total	489	233 (47.6%)	256 (52.4%)

Table S2. The diagnostic value of MRI features for central lymph nodal metastases

MRI Features	Positive LN	Negative LN	*p* Value	OR	95%CI
Long diameter					
lateral	14.584±7.902	11.227±6.195	0.003	-	-
central	8.937±3.955	7.613±4.591	0.428	-	-
Lateral long diameter≥9 mm	0.544	0.784	0.001	3.040	1.550 - 5.965
Short diameter					
lateral	8.056±4.018	6.268±3.309	0.002	-	-
central	5.944±2.439	4.212±1.614	0.069	-	-
Lateral short diameter≥8 mm	0.386	0.189873418	0.005	2.686	1.324 - 5.449
L/S ratiol					
lateral	1.859±0.682	1.838±0.563	0.825	-	-
central	1.722±0.474	1.522±0.381	0.226	-	-
Irregular shape	0.757	0.920	0.002	0.272	0.113 - 5.449
Unsmooth edge	0.870	0.977	0.014	0.157	0.035 - 0.705
Necrosis or cystic degeneration	0.661	0.862	0.001	0.312	0.152 - 0.641
Exaggerated enhancement	0.643	0.874	0.000	0.261	0.125 - 0.547
Fusion	0.217	0.092	0.017	2.743	1.171 - 0.547
Left-right symmetry	0.5	0.609375	0.248	0.641	0.301 - 1.366

Table S3. Nodal compartments detected at US, MRI, US+MRI of the resected specimen, along with the metrics of the diagnostic ability (pathological diagnosis was used as the gold standard method).

		Ture Positive Detected Nodal Levels	Detected Nodal Levels	Additional Malignant Compartments Detected by Modality vs US (%)	Sensitivity (95%CI)	Specificity (95%CI)	PPV (95%CI)	NPV (95%CI)	Accuracy
**Lateral**	US	108	146	-	55.3% (47.4%-63.3%)	83.8% (77.8%-89.7%)	77.6% (69.7%-85.5%)	64.9% (58.2%-71.7%)	69.4%
	MRI	115	148	+ 7/189 (3.7)	59.3% (51.5%-67.2%)	87.2% (81.8%-92.6%)	82.4% (75.2%-89.6%)	67.9% (61.3%-74.5%)	72.5%
	US+MRI	138	193	+ 30/189 (15.8)	71.3% (64.1%-78.6%)	75.7% (68.8%-82.6%)	74.8% (67.7%-81.9%)	72.3% (65.2%-79.3%)	73.2%
II	US	20	27	-	37.8% (22.2%-53.5%)	92.3% (83.9%-100%)	82.4% (64.2%-100%)	61.0% (48.6%-73.5%)	63.8%
	MRI	28	36	+ 8/49 (16.3)	51.4% (35.2%-67.5%)	87.2% (76.7%-97.7%)	79.2% (62.9%-95.4%)	65.4% (52.5%-78.3%)	71.0%
	US+MRI	32	44	+12/49 (24.5)	59.5% (43.6%-75.3%)	82.1% (70.0%-94.1%)	75.9% (60.3%-91.4%)	68.1% (54.8%-81.4%)	71.3%
III	US	36	48	-	62.8% (48.3%-77.2%)	75.8% (61.1%-90.4%)	77.1% (63.2%-91.1%)	61.0% (46.0%-75.9%)	71.1%
	MRI	33	39	- 3/54 (-)	55.8% (41.0%-70.7%)	87.9% (76.7%-99.0%)	85.7% (72.8%-98.7%)	60.4% (46.6%-74.3%)	74.4%
	US+MRI	42	55	+6/54 (11.1)	74.4% (61.4%-87.5%)	72.7% (57.5%-87.9%)	78.0% (65.4%-90.7%)	68.6% (53.2%-84.0%)	75.6%
IV	US	50	62	-	76.9% (65.5%-88.4%)	75.0% (57.7%-92.3%)	87.0% (77.2%-96.7%)	60.0% (42.5%-77.5%)	75.0%
	MRI	48	58	- 2/63 (-)	76.9% (65.5%-88.4%)	83.3% (68.4%-98.2%)	90.9% (82.4%-99.4%)	62.5% (45.7%-79.3%)	75.9%
	US+MRI	58	72	+ 8/63 (12.7)	90.4% (82.4%-98.4%)	70.8% (52.6%-89.0%)	87.0% (78.1%-96.0%)	77.3% (59.8%-94.8%)	79.0%
V	US	2	9	-	22.2% (3.0%-41.4%)	88.5% (79.8%-97.1%)	40.0% (9.6%-70.4%)	76.7% (66.0%-87.4%)	48.9%
	MRI	6	15	+ 4/23 (17.4)	55.6%	90.4%	66.7%	85.5%	56.0%

					(32.6%-78.5%)	(82.4%-98.4%)	(42.8%-90.5%)	(76.1%-94.8%)	
	US+MRI	6	22	+ 4/23（17.4）	66.7% (44.9%-88.4%)	86.5% (77.3%-95.8%)	63.2% (41.5%-84.8%)	88.2% (79.4%-97.1%)	50.5%
	US	21	30	-	43.2% (27.3%-59.2%)	84.2% (72.6%-95.8%)	72.7% (54.1%-91.3%)	60.4% (47.2%-73.5%)	64.7%
**Central**	MRI	37	47	+ 16/44（36.4）	83.8% (71.9%-95.7%)	78.9% (66.0%-91.9%)	79.5% (66.8%-92.2%)	83.3% (71.2%-95.5%)	81.8%
	US+MRI	40	54	+19/44（43.2）	91.9% (83.1%-100%)	73.7% (59.7%-87.7%)	77.3% (64.9%-89.7%)	90.3% (79.9%-100%)	81.2%
	US	129	176	-	52.9% (45.8%-60.1%)	83.9% (78.6%-89.2%)	76.7% (69.5%-84.0%)	63.9% (57.9%-70.0%)	68.5%
**Total**	MRI	152	195	**+23/233（9.8）**	64.2% (57.3%-71.0%)	85.5% (80.4%-90.5%)	81.6% (75.4%-87.9%)	70.4% (64.4%-76.3%)	74.2%
	US+MRI	178	247	**+49/233（21）**	75.4% (69.2%-81.6%)	75.3% (69.1%-81.5%)	75.4% (69.2%-81.6%)	75.3% (69.1%-81.5%)	74.7%

Table S4. Patterns of radiologic–pathologic discordance involving MRI in recurrent/persistent PTC reoperations.

**Characteristics**	**MRI True Positive & US False Negative (n = 23 Levels)**	**MRI False Negative (n = 43 Levels)**	**MRI False Positive (n = 81 Levels)**
Size, mm Mean ± SD	6.824 ± 2.315	5.256 ± 1.894	7.955 ± 2.138
<5 mm, n (%)	8 (34.8)	32 (74.4)	0 (0)
5-10 mm, n (%)	12 (52.2)	11 (25.6)	52 (64.2)
>10 mm, n (%)	3 (13.0)	0 (0)	29 (35.8)
Location, n (%)			
Central	15 (65.2)	28 (65.1)	45 (55.6)
Retropharyngeal	6 (26.1)	5 (11.6)	8 (9.9)
Paratracheal	5 (21.7)	12 (27.9)	15 (18.5)
Lateral (II-V)	8 (34.8)	15 (34.9)	36 (44.4)
Key features	Small, cystic,deep location	Microscopic,<5mm	Reactive hyperplasia
Typical Imaging Features	Round shape,loss of hilum,heterogeneous	Below MRI resolution,minimal enhancement	Rounded, heterogeneous,loss of hilum
Possible Explanation	Acoustic shadow or scar tissue interference on US; deep location	Below spatial resolution limit of MRI	Hashimoto's, reactive changes mimic malignancy

Table S5. Number of Radioactive iodine therapy after Reoperation.

Number of RAI after Reoperation , n (%)	Before Matching			After Matching		
	US Group (n = 299)	US+MRI Group (n = 76)	*p* Value	US Group (n = 103)	US+MRI Group (n = 63)	*p* Value
0	127 (42.47)	30 (39.47)		49 (48.51)	25 (40.32)	
1	116 (38.80)	33 (43.42)		37 (36.63)	28 (45.16)	
2	52 (17.39)	10 (13.16)	0.325	13 (12.87)	7 (11.29)	0.557
3	3 (1.00)	3 (3.95)		1 (0.99)	2 (3.23)	
4	1 (0.33)	0 (0.00)		1 (0.99)	0 (0.00)	
